# Semi‐automated hippocampal avoidance whole‐brain radiotherapy planning

**DOI:** 10.1002/acm2.70076

**Published:** 2025-03-18

**Authors:** Dong Joo Rhee, Subha Perni, Kelly J. Perrin, Kevin E. Casey, Alexandra O. Leone, Callistus M. Nguyen, Laurence E. Court, He Wang, Xin Wang, Eun Young Han

**Affiliations:** ^1^ Department of Radiation Physics The University of Texas MD Anderson Cancer Center Houston Texas USA; ^2^ Department of Central Nervous System Radiation Oncology The University of Texas MD Anderson Cancer Center Houston Texas USA

**Keywords:** autocontouring, autoplanning, hippocampal avoidance WBRT, whole brain radiotherapy

## Abstract

**Background:**

Hippocampal avoidance whole‐brain radiotherapy (HA‐WBRT) is designed to spare cognitive function by reducing radiation dose to the hippocampus during the treatment of brain metastases. Current manual planning methods can be time‐consuming and may vary in quality, necessitating the development of automated approaches to streamline the process and ensure consistency.

**Purpose:**

To automate hippocampal avoidance whole‐brain radiotherapy (HA‐WBRT) planning.

**Methods:**

Our algorithm automatically contours organs‐at‐risk (OARs) and the hippocampal‐avoidance brain target. The algorithm generates planning structures from given contours, utilizing preset beam parameters and dose constraints for optimization. If the dose constraints are unmet, “hotspot” contours will be created to improve dosimetry. The algorithm was written with RayStation's scripting feature and was compared with clinically approved manual HA‐WBRT plans for 20 retrospective patients using target and OAR dose metrics, with statistical analysis performed using the Student's *t*‐test. In the qualitative review, an experienced radiation oncologist blindly scored both the manual plans and autoplans for qualitative review. Lastly, IMRT QA was performed to determine the plans’ deliverability.

**Results:**

The autoplans demonstrated a better target coverage with a more uniform dose. With a prescription dose of 3000 cGy, autoplans achieved higher *D*_95%_ (3026 cGy vs. 2998 cGy, *p* = 0.02) and lower *D*_max_ (3337 cGy vs. 3533 cGy, *p* < 0.01) for the target. The maximum OAR doses were substantially lower in the eyes of autoplans (1727 cGy vs. 2176 cGy, *p* < 0.01), while the other OARs had similar maximum doses to those of the manual plans. The autoplans met all of the in‐house dose constraints, and the minimum dose to the hippocampus was reduced by 5% compared to the manual plans; the average MU was 1376 ± 329 MU for the manual plans and 1141 ± 64 MU for the autoplans. Autoplan generation took an average of 100.2 ± 16.3 minutes (range 62.9–127.9 min). In the qualitative review, the average scores were 4.9 ± 0.4 for the autoplans and 3.4 ± 1.0 for the manual plans. The gamma criteria results for IMRT QA were 96.4 ± 2.1% for the autoplans and 91.6 ± 5.3% for the manual plans.

**Conclusions:**

Our rule‐based autoplanning algorithm produces high‐quality plans that are comparable to those of manual planning, demonstrating autoplanning's potential to reduce HA‐WBRT planning time while ensuring consistent plan quality.

## 1 INTRODUCTION

Whole brain radiotherapy (WBRT) is a common treatment for patients with brain metastases, which are found in approximately 20%–40% of adult cancer patients.^1^, ^2^, ^3^ However, WBRT is associated with neurocognitive toxicity, which can lead to decreased quality of life.^4^

Advances in radiation delivery techniques, such as volumetric modulated arc therapy (VMAT), have led to the development of hippocampal avoidance WBRT (HA‐WBRT). This technique spares the hippocampus, which may contain neural crest progenitor cells, while delivering a uniform dose to the rest of the brain, and has been shown to be associated with decreased decline in neurocognitive function compared to traditional WBRT.^1^, ^5^

Although HA‐WBRT is the preferred treatment for patients with brain metastases, it is significantly more time‐consuming to generate high‐quality HA‐WBRT plans than 3D‐based WBRT plans. However, many candidates for WBRT have symptomatic brain metastases,^6^ making it crucial to create treatment plans in a timely manner. Even for those with asymptomatic brain metastases, radiation planning time can mean additional delays in the initiation or continuation of systemic therapies. Additionally, inter‐planner variability can result in inconsistent plan quality.^7^ To address these issues, various autoplanning approaches for both regular WBRT and HA‐WBRT have been developed and implemented in clinical setttings.^8^, ^9^, ^10^, ^11^, ^12^ However, previous studies have used the dose constraints from the RTOG 0933^5^ or NRG CC001^1^ trials, which did not exclusively limit the dose to other critical structures, such as the brainstem, spinal cord, eyes, and lenses.

Since 2021, automated WBRT planning has been clinically implemented for same‐day treatment using a graphical user interface developed within RayStation (RaySearch Laboratories, Stockholm, Sweden) at our institution. The next logical step is to incorporate HA‐WBRT plans into this automated workflow.

To consistently and efficiently generate HA‐WBRT plans while adhering to the specific dose constraints used at our institution, we developed a semi‐autoplanning algorithm that emulates the techniques of our senior dosimetrists. In this study, we designed algorithms that were capable of automatically creating HA‐WBRT plans that included critical structures, such as optic structures, brainstem, spinal cord, eyes, and lenses. We determined the clinical acceptability of autoplans by comparing their quantitative and qualitative characteristics with these automatically created plans by comparing them with those of manually created plans that had been used in 20 retrospectively treated patients.

## 2 METHODS

We developed an autoplanning system for HA‐WBRT using VMAT in RayStation treatment planning system. The plan was designed to uniformly cover the entire brain while minimizing the dose to the hippocampi. The prescription dose for these plans was 3000 cGy in 10 fractions.

### 2.1 Contouring

To generate and evaluate the HA‐WBRT plans, the target and normal structure contours were initially generated on CT images. For the clinical target volume (CTV), the brain was contoured using our in‐house autocontouring system,^13^ and then reviewed and revised by a physician. The normal structures, including the optic nerves, chiasm, eyes, lenses, brainstem, and spinal cord, were automatically generated using the same autocontouring system. The hippocampi were manually contoured on MR simulation images and then copied to the CT images registered to the MR images.

Once all of the original structures were contoured, extra planning structures were generated on the basis of certain rules. The planning target volume (PTV) was defined as the CTV minus the right and left hippocampus with a 5‐mm expansion, as defined in RTOG 0933.^14^ Additionally, other planning structures, such as the volume between the two hippocampi and the ring structure around the PTV, were automatically contoured to ensure dose coverage in those volumes and achieve a fast dose fall‐off outside of the target area. Detailed information about how the planning structures were generated is provided in Appendix A.

### 2.2 Beam placement and dose goals

In our autoplanning algorithm, we used three full VMAT arcs to generate the plan. The fixing jaws were set to 20 cm in the lateral directions (10 cm on each side) and included a 1‐cm margin above and below the CTV contour in the superior and inferior directions. The maximum gantry spacing was 2°, and collimator angles of 5°, 355°, and 90° were used for each arc.

The dose goals were also defined to quantitatively evaluate the autoplans. For target coverage, the prescription dose (3000 cGy) should cover at least 95% of the PTV, and 2500 cGy should cover at least 98% of the PTV, with *D*_2%_ < 3750 cGy and the maximum dose < 3500 cGy. For the hippocampi, the maximum dose should be <1600 cGy, and the dose to 100% of the hippocampi should be <900 cGy. For the optic nerves, chiasm, brainstem, and spinal cord, the maximum dose should be <3300 cGy. For the eyes and lenses, the maximum doses should be <2000 and 500 cGy, respectively.

### 2.3 Autoplanning algorithm

**FIGURE 1:**
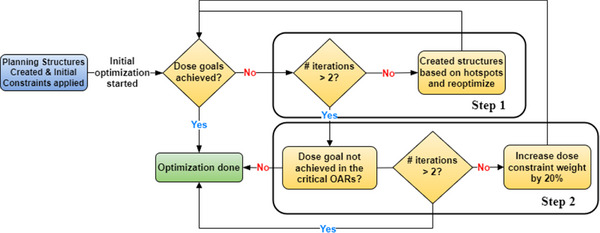
Workflow of the HA‐WBRT autoplanning algorithm. The planning structures and beam configurations were determined, and then initial optimization was performed. Dose goals were evaluated on the final plan. If the dose goals were not met, the dose constraints on the hotspots were added and the dose constraints on normal structures were adjusted for further optimization.

Once all of the contours and initial beam parameters had been automatically generated, the autoplanning algorithm began optimizing the plan. The automatic optimization process was developed to mimic our dosimetrists’ approach to generating HA‐WBRT plans, as demonstrated in Figure 1. First, the algorithm runs the optimization algorithm with 200 iterations. Next, the plan is normalized so that at least 96% of the PTV is covered by the prescription dose. After plan normalization, doses higher than 3300 cGy within the target or the normal structures are identified as “hotspot” structures and dose constraints for these structures are added and re‐optimized. This is repeated twice unless all of the dose goals are met beforehand (Step 1).

If all of the dose goals are not met after Step 1, the dose constraint weights are increased by 20% for the normal structures that do not meet the dose goals, and re‐optimization is performed. This step is repeated twice unless all of the dose goals are met beforehand (Step 2). As the plan is always normalized to achieve target coverage, we can focus on the normal structure dose constraints during the optimization process. Once all of the dose goals were met or the number of iterations exceeded the pre‐determined value, the optimization process was complete. Further details are provided in Appendix A.

### 2.4 Evaluation and comparison of the autoplans and manual plans

Radiotherapy plans were automatically generated for 20 retrospective patients who had been treated with manual HA‐WBRT plans at MD Anderson Cancer Center from June 1, 2020 to July 31, 2023. The manual plans were designed to achieve the same dosimetric goals described in Section 2.2. We compared the quantitative dose metrics, including the dose goals and multiple target coverage parameters, between the manual plan and autoplan pairs using Student's *t*‐test, applying a *p* < 0.05 to consider a statistically significant difference between the pairs.

For the qualitative evaluation, an experienced radiation oncologist at MD Anderson Cancer Center conducted a blind review of both the autoplans and manual plans. The radiation oncologist assessed the plans using a 5‐point Likert scale, as defined in Table 1. Plans that scored ≥3 were considered clinically acceptable.

**TABLE 1:** Likert scale for scoring automatically generated radiotherapy plans.

Score		Description
**5**	**Strongly agree**	Use as‐is. Clinically acceptable. Plans can be used for treatment without change.
**4**	**Agree**	Minor edits that are not necessary. Stylistic changes are preferred, but not clinically important. Current plans are clinically acceptable.
**3**	**Neither agree nor disagree**	Minor edits that are necessary. Minor edits are those that can be made in less time than starting from scratch or are expected to have minimal effect on treatment outcome.
**2**	**Disagree**	Major edits. Necessary edits are required to ensure appropriate treatment and are sufficiently significant that the user would prefer to start from scratch.
**1**	**Strongly disagree**	Unusable. Quality of the automatically generated plans is so bad that they are unusable.

Furthermore, we ran Mobius (Varian Medical Systems, Inc., Palo Alto, CA, USA) for patient‐specific QA on both the manual plans and autoplans to assess the plan deliverability. We used a gamma passing rate ≥90% with 3%/2 mm criteria and a 10% dose threshold, as recommended in TG‐218.^15^

## 3 RESULTS

Quantitative and qualitative analyses were performed to compare the autoplans and manual plans.

### 3.1 Quantitative comparison

Important dose metrics were compared between the manual plans and autoplans on the quantitative analysis. For the target (manual vs. automatic), the values were 2733 ± 104 cGy versus 2698 ± 35 cGy for *D*_98%_ (*p* = 0.20), 2998 ± 48 cGy versus 3026 ± 14 cGy for *D*_95%_ (*p* = 0.02), 3302 ± 44 cGy versus 3274 ± 17 cGy for *D*_2%_ (*p* = 0.02), and 3533 ± 93 cGy versus 3337 ± 23 cGy for maximum dose (*p* < 0.01), respectively. The homogeneity indices (HI), defined as (*D*_2%_ − *D*_98%_) / *D*_p_ × 100, where *D*_p_ is the prescription dose, were 19.0 for manual plans and 19.2 for autoplans.

**FIGURE 2:**
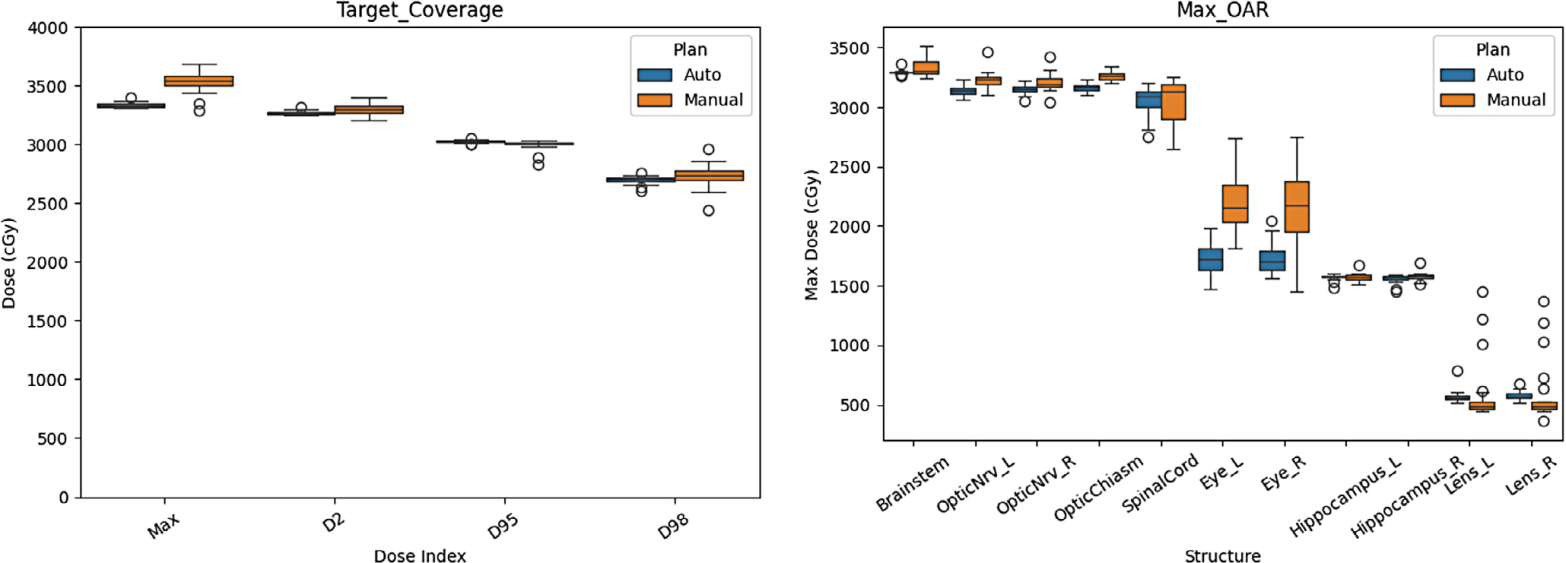
Boxplots for target and maximum OAR doses. The maximum, *D*_2%_, *D*_95%_, and *D*_98%_ doses for the targets, along with the maximum doses for the OARs, were plotted for both manual plans and autoplans.

For normal structures, the maximum doses for the manual plans versus autoplans were 3332 ± 72 cGy versus 3292 ± 17 cGy for the brainstem (*p* = 0.02), 3223 ± 76 cGy versus 3145 ± 42 cGy for the optic nerves (*p* < 0.01), 3261 ± 35 cGy versus 3169 ± 36 cGy for the chiasm (*p* < 0.01), 3050 ± 195 cGy versus 3047 ± 136 cGy for the spinal cord (*p* = 0.93), 2176 ± 306 cGy versus 1727 ± 136 cGy for the eyes (*p* < 0.01), 1576 ± 35 cGy versus 1565 ± 32 cGy for the hippocampi (*p* = 0.14), and 600 ± 275 cGy versus 575 ± 48 cGy for the lenses (*p* = 0.58). The minimum dose (*D*_100%_) to the hippocampus was 894 ± 65 cGy versus 850 ± 22 cGy (*p* < 0.01). The boxplots for these distributions are shown in Figure 2.

Autoplan generation took 100.2 ± 16.3 min on average (range 62.9–127.9 min). The average MU was 1376 ± 329 MU for the manual plans and 1141 ± 64 MU for the autoplans (300 cGy fraction dose). For IMRT QA results with Mobius, the average gamma values were 96.4 ± 2.1% for the autoplans and 91.6 ± 5.3% for the manual plans with 3%/2 mm criteria.

### 3.2 Qualitative comparison

**FIGURE 3:**
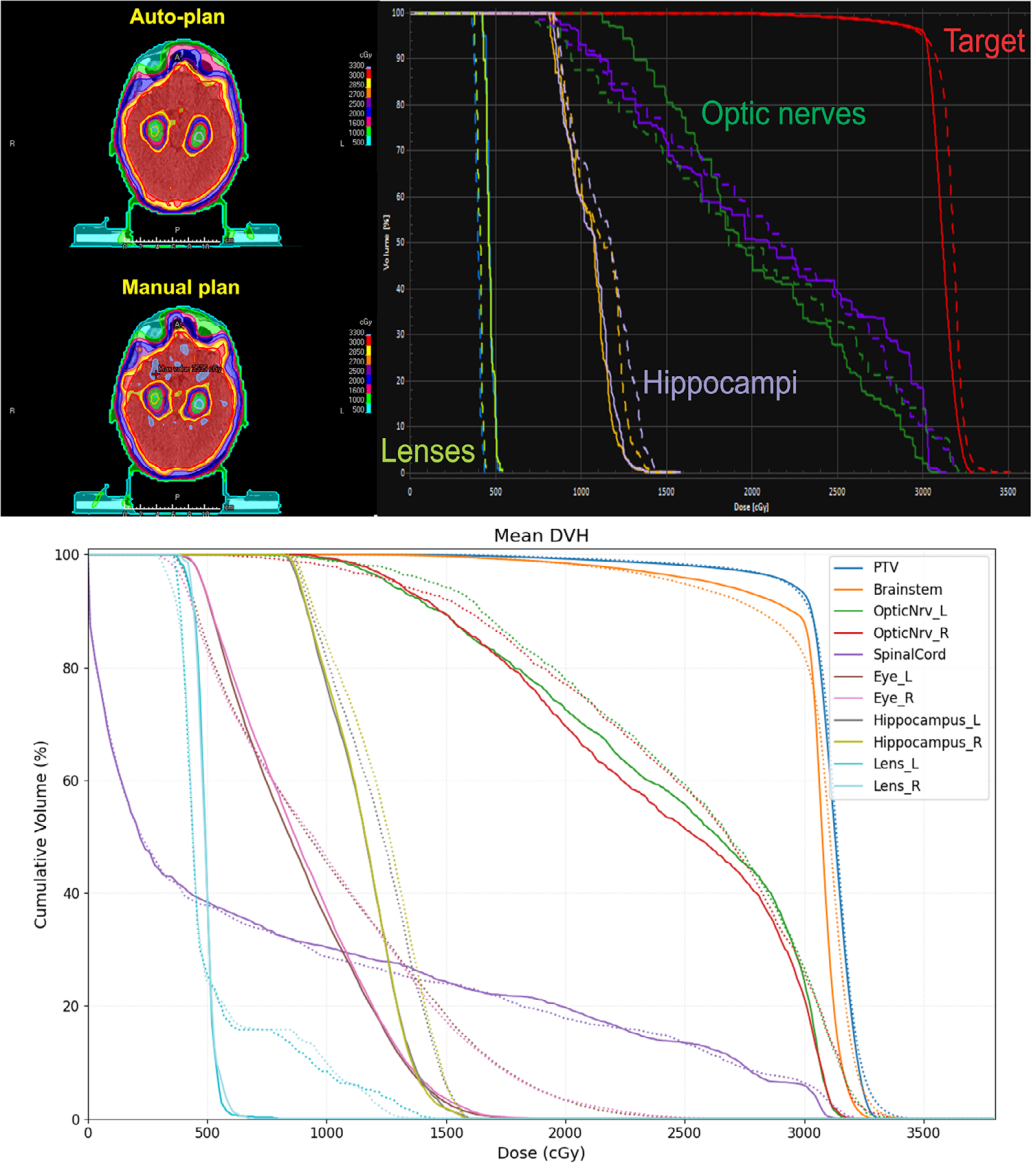
**Dose distributions and DVH for autoplans and manual plans**. An example of the dose distribution of autoplans and manual plans, alongside DVH curves, for the same patient. The autoplans exhibited a more uniform dose distribution, with fewer hotspots (indicated by light purple) within the target area. In the DVH, the autoplans (represented by solid lines) and manual plans (represented by dashed lines) show similar dose distributions for OARs, while the autoplans demonstrate improved target coverage. Similarly, the bottom figure illustrates the mean DVH for each organ for both the autoplans (solid lines) and manual plans (dashed lines), demonstrating improved target coverage and lower OAR doses.

Both manual and autoplans from 20 retrospective patients were blindly reviewed and scored by an experienced radiation oncologist at MD Anderson Cancer Center (Figure 3), and the scores of the 20 retrospectively selected patient plans are presented in Table 2. For the autoplans, the average score was 4.9/5.0, with all plans being clinically acceptable. One autoplan received a score of 3 because the brainstem maximum dose was 3380 cGy, which exceeded the dose goal of 3300 cGy by 80 cGy (2.5%). For the manual plans, the average score was 3.4/5.0. Of the 20 plans, 16 were clinically acceptable; the remaining 4 required major revision. Most scores of 2 or 3 were due to exceeding the maximum dose criteria for the brainstem and CTV inhomogeneity (i.e., multiple hotspots within the CTV).

**TABLE 2:** Physician scoring results for autoplans and manual plans for 20 patients.

	# of plans for each score				
Treatment technique	5	4	3	2	1
**Autoplans**	19	0	1	0	0
**Manual plans**	4	3	9	4	0

## 4 DISCUSSION

On the quantitative analysis, the target coverage was similar between the autoplans and manual plans, while the maximum doses to the targets were significantly better for the autoplans. For the normal structures, the maximum doses were significantly lower for the eyes, brainstem, optic nerves, and chiasm, while the maximum doses for the rest of the OARs were similar. The minimum dose to the hippocampus was 5% lower than the manual plans. These findings demonstrate that our autoplanning algorithm can effectively reduce hotspots in the target and achieve reasonable target coverage while meeting all of the dose goals. It also tends to make the dose to the target more uniform than do human planners. Furthermore, the MU for the autoplan was substantially lower than those for the manual plan (1141 MU vs. 1376 MU) on average, which can slightly reduce delivery time and plan complexity. Additionally, this lower MU likely resulted in better IMRT QA outcomes (96.4% vs. 91.6%) for the autoplans, as they were less modulated than were the manual plans.

On the qualitative analysis, all of the autoplans were found to be clinically acceptable without modification (score ≥ 3). Some of the manual plans required minor or major modifications because the maximum dose constraints for the brainstem exceeded our criteria (3300 cGy) due to slight variation on the brainstem dose constraint among physicians. Furthermore, although the autoplans achieved substantially better dose homogeneity within the CTV, the dose distribution within the CTV for the manual plans was visually unsatisfactory (hotter), despite meeting all the required target dose goals. We believe that this contributed to the relatively low scores in the manual plan evaluations.

Our autoplanning algorithm takes about 2 h to generate a plan without any human involvement, which is substantially shorter than the time required for manual plans, which take about a day in our clinic. Since many candidates for HA‐WBRT are emergency patients, it is crucial to reduce the time from simulation to treatment to maximize treatment outcomes and minimize patient inconvenience. Although the planning process is being automated and streamlined to minimize delays, the next biggest bottleneck is the need for both CT and MR simulations in these patients. The hippocampi must first be contoured on MR images, as they are not clearly distinguishable on CT simulation images. Consequently, MR simulation is mandatory for delivering HA‐WBRT in our clinic. However, acquiring both CT and MR simulations requires additional time and cost. Fortunately, recent advancements in AI allow us to generate synthetic CT images from MR simulations,^16^, ^17^, ^18^, ^19^ which can provide electron density information for accurate dose calculations. For future studies, we are developing an autocontouring system for hippocampi in MR images and working to automate the entire planning process for same‐day treatment, from MR simulation to plan generation. This includes creating synthetic CT images from MR data, autocontouring all structures, and generating high‐quality HA‐WBRT plans.

A key advantage of autoplanning is the streamlined and standardized workflow that reduces the reliance on multiple manual steps and back‐and‐forth communication among the treatment team. By cutting down on wait times for tasks such as physician contour review and dosimetrist adjustments, autoplanning allows for faster plan generation, which is particularly beneficial for emergency cases. Additionally, this approach conserves valuable human resources and mitigates the impact of operator variability. As a result, facilities can expect more consistent plan quality, improved utilization of staff expertise, and ultimately a more efficient clinical workflow.

## 5 CONCLUSIONS

We demonstrated that our autoplanning system, combined with an autocontouring system, can generate clinically acceptable plans for HA‐WBRT within 2 h. The autoplan quality was equivalent to or better than the manual plans in both the quantitative and qualitative assessments. This semi‐autoplanning algorithm can be implemented as a same‐day treatment modality in the future as it can save a significant amount of dosimetrists' time in planning.

## AUTHOR CONTRIBUTIONS

**Dong Joo Rhee**: Conceptualized and designed the study; developed the autoplanning algorithm; supervised data analysis; and wrote the initial draft of the manuscript. **Subha Perni**: Reviewed and scored the automatic and manual plans for 20 patients and provided contour reviews. **Kelly J. Perrin**: Designed the study and supervised the development and review of the autoplanning algorithm. **Kevin E. Casey**: Collected and analyzed IMRT QA data for both automatic and manual plans. **Alexandra O. Leone and Callistus M. Nguyen**: Collected and curated patient data and generated the necessary contours for autoplanning. **Laurence E. Court, He Wang, and Xin Wang**: Supervised data analysis and critically revised the manuscript for important intellectual content. **Eun Young Han**: Conceptualized and designed the study and critically revised the manuscript.

## CONFLICT OF INTEREST STATEMENT

The authors declare no conflicts of interest.

## APPENDIX A

### CONTOURING AND AUTOPLANNING WORKFLOWS

Contouring workflow

1. Load patient CT and MR images.
2. Generate initial contours using the autocontouring system to segment critical structures (hippocampi, brainstem, spinal cord, optic structures, etc).
3. Manually delineate hippocampi on MR images and register MR contours to CT images.
4. Generate planning structures automatically:
  a. Create PTV by subtracting the hippocampi with a 5-mm margin from CTV.
  b. Add auxiliary planning structures:
    b.1. Create a “Ring” contour by (PTV + 5 mm) PTV.
    b.2. Create a “Normal Tissue Avoidance” contour by Body (PTV + Hippocampi). Remove contours that extend more than 1.5 cm below the most inferior portion of the PTV. Both Ring and Normal Tissue Avoidance contours are designed to achieve a sharp dose fall‐off.
    b.3. Create an “Outer Hippocampi Push” contour by Hippocampi (Hippocampi with a 1 mm contraction). This will create outer rinds for each hippocampus to push the maximum dose of 16 Gy away.
    b.4. Create a “Target near Hippocampi” contour by first creating a hippocampi shell (hippocampi with 1 cm superior‐inferior, 2 cm anterior, 1.5 cm posterior, and left‐right margins) and subtracting the hippocampi with a 1 cm margin. This represents the PTV near the hippocampi and ensures adequate coverage in these areas.

Autoplanning workflow

1. Initialize dose optimization with predefined constraints.
  a. Dose constraints for the target and OARs are based on the dose goals in section 2.2.
  b. Dose constraints for the planning structures are as follows:
    Ring: Dmax < 2880 cGy
    Normal Tissue Avoidance: Dmax < 1900 cGy
    Outer Hippocampal Push: Dmax < 1250 cGy
    Target near Hippocampi: Dmin > 3000 cGy
2. Normalize the plan such that 96% of the PTV receives the prescription dose.
3. Identify hotspots:
  a. Locate areas where the dose exceeds defined thresholds (e.g., > 3300 cGy).
  b. Create hotspot structures.
4. Re-optimize plan:
  a. Add constraints to reduce the dose to hotspot structures.
  b. Increase the weight of existing constraints by 20% if dose thresholds are not met.
5. Repeat steps 3 and 4 up to two iterations or until all dose goals are satisfied.
6. Finalize and save the optimized plan.
